# The impact of nurses’ caring behaviors and personality traits on workplace violence

**DOI:** 10.3389/fpsyg.2025.1576252

**Published:** 2025-06-18

**Authors:** Hongjuan Chang, Xin Liu, Mengmeng Hu, Rui Zeng, Chun Zhang, Huanhuan Luo

**Affiliations:** ^1^School of Medicine, Wuhan University of Science and Technology, Wuhan, China; ^2^School of Medicine, Tarim University, Alaer, Xinjiang, China

**Keywords:** nurses, caring, personality traits, tendency score matching, impact

## Abstract

**Aims:**

Based on the propensity score matching method, this study analyzes the correlation between nurses’ exposure to workplace violence, caring behaviors, and personality traits. The analysis provides a foundation for developing individualized strategies to prevent workplace violence among nurses.

**Methods:**

Convenience sampling was conducted from February to June 2024 in Grade 3A hospitals across 11 Chinese provinces. The study utilized the Workplace Violence Frequency Measurement Scale, Caring Behaviors Inventory, and Ten-Item Personality Inventory for data collection. The analysis employed propensity score matching to pair nurses experiencing low-frequency workplace violence with those experiencing high-frequency workplace violence in a 1:2 ratio, controlling for confounding variables. The associations between personality traits, caring behaviors, and workplace violence were then analyzed.

**Results:**

Following propensity score matching to control for baseline information, the analysis included 622 nurses (403 in the low-frequency exposure group and 219 in the high-frequency exposure group). The findings revealed respect and connectedness (*OR* = 0.946, 95.0% *CI*: 0.912 ~ 0.981), emotional stability (*OR* = 0.814, 95.0% *CI*: 0.711 ~ 0.906) as negative predictive effects, and openness as a positive predictive effect (*OR* = 1.250, 95.0% *CI*: 1.065 ~ 1.398).

**Conclusion:**

Nurses demonstrating patient respect and maintaining close contact, along with those exhibiting emotional stability, experience low-frequency exposure to workplace violence; conversely, nurses with more open personalities experience higher frequency exposure. These findings suggest the importance of enhancing nurses’ caring behaviors and implementing personality-specific training programs to address workplace violence.

## 1 Introduction

Workplace violence (WPV) encompasses incidents where healthcare professionals experience verbal abuse, threats, or physical assaults during their occupational duties, presenting explicit or implicit threats to their safety, well-being, and health (Al-Qadi, 2021). Traditionally, WPV within healthcare professionals is classified as physical and psychological violence (Shi et al., 2020). It is reported that the majority of healthcare professionals are exposed to physical and sexual violence, especially verbal violence (Demirci and Uğurluoğlu, 2020). A recent survey from China indicates that 62.4% of healthcare professionals experienced WPV in 2020 (Cai and Chen, 2024). Among healthcare professionals, nurses experience the highest exposure to WPV (Demirci and Uğurluoğlu, 2020; Doehring et al., 2024). WPV against nurses has emerged as a critical public safety concern in healthcare. Such violence can inflict physical harm on affected nurses (Lee and Lee, 2022) and negatively impact their work performance (Liu et al., 2019a), job satisfaction (Xie et al., 2023), job stress (Wang et al., 2022), burnout (Fei et al., 2023), and turnover intention (Kang et al., 2020). Consequently, investigating the factors influencing WPV against nurses is crucial for developing effective preventive strategies.

Previous research has examined WPV influencing factors among healthcare professionals broadly, demonstrating that demographic characteristics including gender, age, professional title, and position significantly correlate with WPV incidence (Niu et al., 2019; Cheung and Yip, 2017). Moreover, research indicates that nurses’ WPV experiences are shaped not only by external factors such as department characteristics and patient features but also by nurses’ individual traits (Chen et al., 2024; Serpici et al., 2024). Personality traits, characterized as stable and enduring internal characteristics manifested through behavioral patterns, attitudes, emotions, and habits (Ghafouri et al., 2021), are fundamental in nursing practice. Studies reveal that nurses with varying personality traits experience different levels of WPV (Serpici et al., 2024). Research has shown that low levels of vulnerability trait (neuroticism) correlate with WPV experience (Nøland et al., 2021). Additionally, Nurse caring has long been regarded as critical core professional value of compassion and an ability to respond with humanity and kindness to others’ pain, distress, anxiety, or needs (Loke et al., 2015). Research indicates that nurses demonstrating caring behaviors are less likely to experience aggression, suggesting that a safe working environment correlates with nurses’ caring behaviors. However, limited research has examined the relationships between WPV, personality traits, and caring behaviors while accounting for potential external factors. Given possible confounding bias from varying external factors, these relationships warrant investigation with appropriate controls.

Propensity score matching (PSM) represents a statistical methodology for analyzing observational study data (Chen et al., 2022). This approach reduces data bias and confounding variable effects, enabling more accurate comparisons between exposure and control groups (Ding et al., 2023). Therefore, this study introduces PSM to match baseline socio-demographic characteristics, thereby enabling a rigorous exploration of the associations among WPV, personality traits, and caring behaviors.

## 2 Materials and methods

### 2.1 Study design and setting

This is a cross-sectional observational study carried out with self-reported questionnaires. The study was conducted from February to June 2024. The study was conducted in Grade 3A hospitals in 11 provinces of China, with a total of 3,278 questionnaires collected. The research adhered to the fundamental ethical principles in the Declaration of Helsinki. It was approved by the Ethics Committee of the Wuhan University of Science and Technology School of Medicine (2023091).

### 2.2 Participants

The convenience sampling method was applied to select samples. Inclusion criteria: (1) Working as a clinical nurse in the hospital; (2) obtaining and registering the “nursing license”; (3) working for more than 1 year; (5) voluntarily participating in this survey. Exclusion criteria: (1) nurses who were not from the hospital, including clinical nursing students, non-nursing staff and visiting nurses for advanced training; (2) nurses who were on vacation, retired and absent for work during the questionnaire period. This study was based on obtaining written informed consent from the respondents.

### 2.3 Data collection

Hospital administrators were contacted by members of the project team and trained centrally prior to the survey. During the data collection process, the investigator informed the study participants of the purpose and significance of the study, instructed the nurses to sign the informed consent form, and distributed the electronic questionnaire. Once the nurses completed the questionnaire, it was exported and checked for completeness. A total of 3,290 questionnaires were distributed in this study, and 3,278 valid questionnaires were returned, with a valid recovery rate of 99.6%. Among the effective samples, there are 1,564 individuals in the low-frequency group (47.7%), 1,472 individuals in the medium-frequency group (44.9%), and 242 individuals in the high-frequency group (7.4%). There are no individuals in the zero-frequency group.

### 2.4 Measures

#### 2.4.1 General information questionnaire

The researcher designed a survey that included nurses’ general demographic information (gender, age, marital status, etc.) and career-related information (title, position, years of service).

#### 2.4.2 Workplace violence frequency measurement scale

The scale, developed by Wang (2006), measures and assesses the frequency of workplace violence (WPV) over the past year. The scale comprises five items assessing different dimensions of WPV: physical assault, emotional abuse, threats of intimidation, verbal sexual harassment, and physical sexual harassment. The scoring system is as follows: 0 = no violence, 1 = once, 2 = two or three times, 3 = four or more times, with the total scale score representing the cumulative value of individual entries. Frequency classifications are: zero frequency (score of 0), low frequency (score of 1–5), medium frequency (score of 6–10), and high frequency (score of 11–15). Higher scores indicate increased WPV risk. The Cronbach’s α for this questionnaire in the present study was 0.98.

#### 2.4.3 Caring behaviors inventory

The scale was developed by Wolf (1981) and subsequently revised in 2006 (Wu et al., 2006). The Chinese translation and validation were completed by Da et al. (2017). The scale employs a 6-point Likert format, where participants indicate frequency of occurrence based on their experience (1 = never, 2 = almost never, 3 = occasionally, 4 = often, 5 = almost always, 6 = always). Scores for individual dimensions and the total scale were aggregated, with higher scores indicating superior patient care delivery by nurses. The Cronbach’s α for this questionnaire in the current study was 0.93.

#### 2.4.4 Ten-item personality inventory

The scale was developed by Li (2013) to measure the Big Five factors of personality. It includes five dimensions: extroversion, pleasantness, diligence (rigor), emotional stability (neuroticism), and openness. There are a total of 10 items, and each item is scored on a scale from 1 (absolutely disagree) to 7 (absolutely agree). Each dimension is scored independently, with higher scores indicating more pronounced tendencies in that personality trait. The Cronbach’s α for this questionnaire in this study was 0.93.

### 2.5 Data management and analysis

Data analysis was conducted using R version 3.2 and SPSS27.0. Initially, propensity score matching (PSM) analysis matched baseline socio-demographic characteristics (Kane et al., 2020), including age, sex, marital status, department, professional title, departmental position, educational level, nursing experience duration, employment status/contract type, average monthly income, sleep quality, health status, WPV-related training participation, and hospital interpersonal communication training participation. The study designated nurses experiencing high-frequency WPV as the experimental group and those with low-frequency WPV as the control group. Nearest neighborhood matching was implemented (caliper = 0.2) with a 1:2 ratio (case:control) (Austin, 2011). Subsequently, descriptive statistics analyzed baseline information for both groups before and after matching. Finally, variables were incorporated into a logistic regression model to examine correlations between nurses’ WPV exposure, caring behaviors, and personality traits. The test level is α = 0.05.

## 3 Results

### 3.1 Demographic

Before propensity score matching, significant differences (*p* < 0.05) existed between groups regarding department, title, nursing experience duration, monthly night shift frequency, sleep quality, health status, WPV-related training participation, and hospital interpersonal communication training participation. After matching implementation, no significant between-group differences remained in general information (*p* > 0.05). The follow-up analysis included 622 nurses (403 with low-frequency WPV and 219 with high-frequency WPV) (see Table 1).

**Table 1:** Comparison of general information between the group with low frequency of violence and the group with high frequency of violence before and after matching on the propensity score.

Groups	Pre-matching				Post-matching			
	Low-frequency group (*N* = 1,564)	High-frequency group (*N* = 242)	*t*/χ^2^	*p*	Low-frequency group (*n* = 403)	High-frequency group (*n* = 219)	*t*/χ^2^	*p*
Age	30.92 ± 7.62	31.12 ± 6.49	−0.44	0.661	31.33 ± 6.89	30.94 ± 6.49	0.70	0.487
Sex								
Male	105 (85.4)	18 (14.6)	0.17	0.677	22 (64.7)	12 (35.3)	0.00	0.991
Female	1,459 (86.7)	224 (13.3)			381 (64.8)	207 (35.2)		
Marital status								
Non-married	636 (88.1)	86 (11.9)	6.61	0.072	135 (62.5)	81 (37.5)	1.26	0.808
Wedlock	909 (85.9)	149 (14.1)			257 (65.9)	133 (34.1)		
Dissociation	14 (70.0)	6 (30.0)			10 (66.7)	5 (33.3)		
Pouse	5 (83.3)	1 (0.17)			1 (100)	0		
Department								
Nternal medical	483 (87.3)	70 (12.7)	80.90	<0.001	132 (66.0)	68 (34.0)	4.33	0.749
Surgery	351 (90.0)	39 (10.0)			89 (70.1)	38 (29.9)		
Department of obstetrics and gynecology	99 (95.2)	5 (4.8)			10 (66.7)	5 (33.3)		
Pediatrics	65 (92.9)	5 (7.1)			13 (72.2)	5 (27.8)		
Intensive care unit	128 (83.7)	25 (16.3)			37 (61.7)	23 (38.3)		
Operating room	125 (99.2)	1 (0.8)			2 (66.7)	1 (33.3)		
Emergency department	91 (67.9)	43 (32.1)			44 (60.3)	29 (39.7)		
Others	222 (80.4)	54 (19.6)			76 (60.3)	50 (39.7)		
Professional title								
Nurses	571 (90.1)	63 (9.9)	16.33	<0.001	104 (62.7)	62 (37.3)	0.55	0.909
Senior nurse	523 (82.5)	111 (17.5)			189 (65.9)	98 (34.1)		
Supervisor nurse	381 (87.0)	57 (13.0)			93 (65.5)	49 (34.5)		
Deputy chief nurse and above	89 (89.0)	11 (11.0)			17 (63.0)	10 (37.0)		
Position in department								
General nurses	141 (86.8)	215 (13.2)	0.45	0.797	375 (66.0)	193 (34.0)	4.41	0.111
Head nurse	81 (85.3)	14 (14.7)			16 (53.3)	14 (46.7)		
Others	72 (84.7)	13 (15.3)			12 (50.0)	12 (50.0)		
Educational level								
Secondary	59 (93.7)	4 (6.3)	6.77	0.072	8 (66.7)	4 (33.3)	0.23	0.992
Junior college	466 (88.8)	59 (11.2)			103 (65.2)	55 (34.8)		
Undergraduate	1,030 (85.3)	178 (14.7)			290 (64.6)	159 (35.4)		
Master’s and above	9 (90.0)	1 (10.0)			2 (66.7)	1 (33.3)		
Years of nursing experience								
0–1	244 (94.6)	14 (5.4)	24.15	<0.001	24 (63.2)	14 (36.8)	0.38	0.984
2–5	381 (84.7)	69 (15.3)			110 (63.2)	64 (36.8)		
6–10	387 (82.3)	83 (17.7)			138 (65.4)	73 (34.6)		
11–15	315 (87.3)	46 (12.7)			82 (66.1)	42 (33.9)		
≥16	237 (88.8)	30 (11.2)			49 (65.3)	26 (34.7)		
Employment status								
Official compilation	528 (87.1)	78 (12.9)	0.31	0.856	118 (62.4)	71 (37.6)	0.66	0.718
Personnel agency	174 (87.0)	26 (13.0)			42 (65.6)	22 (34.4)		
Contract employment	862 (8 6.2)	138 (13.8)			243 (65.9)	126 (34.1)		
Average monthly income								
<4,000	234 (87.0)	35 (13.0)	1.41	0.842	64 (67.4)	31 (32.6)	0.83	0.935
4,000–6,000	528 (85.3)	91 (14.7)			142 (62.8)	84 (37.2)		
6,000–8,000	444 (87.4)	64 (12.6)			113 (65.3)	60 (34.7)		
8,000–10,000	230 (87.5)	33 (12.5)			49 (64.5)	27 (35.5)		
>10,000	128 (87.1)	19 (12.9)			35 (64.5)	17 (35.5)		
Monthly frequency of night shifts								
0	494 (92.5)	40 (7.5)	64.43	<0.001	73 (65.2)	39 (34.8)	0.47	0.977
1–4	445 (91.2)	43 (8.8)			86 (66.7)	43 (33.3)		
5–8	430 (82.1)	94 (17.9)			152 (64.7)	83 (35.3)		
9–12	158 (75.2)	52 (24.8)			77 (62.6)	46 (37.4)		
>12	37 (74.0)	13 (26.0)			15 (65.2)	8 (34.8)		
Sleep quality								
Very good	134 (91.8)	12 (8.2)	118.53	<0.001	22 (64.7)	12 (35.3)	0.99	0.910
Good	274 (95.1)	14 (4.9)			32 (69.6)	14 (30.4)		
General	839 (89.9)	94 (10.1)			172 (64.9)	93 (35.1)		
Difference	220 (75.9)	70 (24.1)			115 (65.3)	61 (34.7)		
Very poor	97 (65.1)	52 (34.9)			62 (61.4)	39 (38.6)		
Health status								
Very good	186 (94.9)	10 (5.1)	144.72	<0.001	17 (63.0)	10 (37.0)	1.05	0.902
Good	497 (94.1)	31 (2.9)			54 (63.5)	31 (36.5)		
General	789 (84.8)	141 (15.2)			259 (65.6)	136 (34.4)		
Difference	81 (65.9)	42 (34.1)			62 (65.3)	33 (34.7)		
Very poor	11 (37.9)	18 (62.1)			11 (55.0)	9 (45.0)		
Participation in training related to workplace violence								
Often	243 (93.5)	17 (6.5)	22.86	<0.001	29 (64.4)	16 (35.6)	0.30	0.961
Sometimes	496 (87.9)	68 (12.1)			126 (66.3)	64 (33.7)		
Infrequent	484 (86.3)	77 (13.7)			130 (64.4)	72 (35.6)		
Never	341 (81.0)	80 (19.0)			118 (63.8)	67 (36.2)		
Participation in interpersonal communication training within the hospital								
Often	498 (91.5)	46 (8.5)	28.50	<0.001	100 (69.0)	45 (31.0)	1.50	0.682
Sometimes	639 (87.3)	93 (12.7)			156 (63.7)	89 (36.3)		
Infrequent	329 (80.8)	78 (19.2)			114 (63.0)	67 (37.0)		
Never	98 (79.7)	25 (20.0.3)			33 (64.7)	18 (35.3)		

### 3.2 Univariate analysis of nurses’ caring behaviors and personality traits

The Mann–Whitney U test was applied to the PSM-matched dataset. The analysis included three caring behavior dimensions (respect and connection, knowledge and skills, support and reassurance) and five Big Five personality dimensions (extroversion, agreeableness, dutifulness, emotional stability, and openness). Results revealed significant between-group differences (*p* < 0.05) in respect and connection, knowledge and skills, support and reassurance, emotional stability (neuroticism), and openness (see Table 2).

**Table 2:** Univariate analysis of nurses’ caring behaviors and personality traits.

Item	Low-frequency group (*n* = 403)	High-frequency group (*n* = 219)	*Z*	*P*
Respect and connection	49.02 ± 10.08	46.40 ± 9.58	−3.674	<0.001
Knowledge and technique	25.78 ± 5.04	24.92 ± 5.12	−2.043	0.041
Support and guarantee	46.04 ± 9.09	44.63 ± 9.05	−2.197	0.028
Extraversion	8.38 ± 1.83	8.50 ± 1.78	−0.891	0.373
Agreeableness	10.13 ± 2.11	9.91 ± 2.03	−1.130	0.259
Conscientiousness	9.81 ± 2.17	9.69 ± 2.06	−0.586	0.558
Emotional stability	9.47 ± 2.03	8.93 ± 1.93	−2.604	0.009
Openness	8.88 ± 1.68	9.08 ± 1.69	−2.060	0.039

### 3.3 Logistic regression of nurses’ caring behaviors and personality traits

A binary logistic regression model was constructed using violence frequency as the dependent variable, with Big Five personality traits (emotional stability and openness) and caring behaviors (respect and connection, knowledge and skills, support and assurance) as independent variables, based on univariate analysis results. The variables entering the regression equation were respect and connection, emotional stability, and openness. Results indicated that respect and connection and emotional stability functioned as negative predictors, while openness served as a positive predictor (*p* < 0.05) (see Table 3).

**Table 3:** Logistic regression of nurses’ caring behaviors and personality traits.

Item	β	SE	Wald χ^2^	OR	95%*CI*	*P*
Respect and connection	−0.55	0.019	8.901	0.946	0.912 ~ 0.981	0.003
Emotional stability	−0.206	0.054	14.612	0.814	0.732 ~ 0.904	<0.001
Openness	0.223	0.062	13.082	1.250	1.108 ~ 1.411	<0.001

## 4 Discussion

This study examined the relationship between personality traits and caring behaviors on workplace violence through a cross-sectional survey utilizing a propensity score matching method. Few studies have employed PSM as a statistical approach to investigate the connection between personality traits, caring behaviors, and WPV in nurses. The findings reveal that the frequency of WPV experienced by nurses is significantly affected by factors including respect and connection, emotional stability, and openness (*p* > 0.05).

Previous studies have established a significant correlation between enhanced patient care by nurses and a reduced incidence of WPV (Zhong et al., 2021; Yao et al., 2021). Building on these findings, this study investigates which specific caring behaviors of nurses correlate with WPV occurrence. The analysis demonstrates that nurses who respect patients and establish meaningful connections experience significantly fewer WPV incidents. Some causes that may explain this phenomenon are, first of all, nurses who demonstrate respect and build strong connections establish trust and positive relationships, potentially reducing WPV (Hynnekleiv et al., 2024); Furthermore, patients receiving attentive, informative, and emotional support from nurses tend to feel more respected and understood, leading to reduced aggressive and violent behaviors (Ryan et al., 2022). Additionally, implementing caring communication practices that emphasize proactive inquiry, responsive care, and open dialog helps reduce patient dissatisfaction and misunderstanding, thereby lowering WPV risk (McGilton et al., 2012). These findings align with Hou Yongchao’s research, which indicated that patients may resort to violent behavior when feeling neglected (Hou et al., 2022). Additionally, other studies revealed also identified that WPV-related factors include communication gaps between nurses and patients (Benning et al., 2024). Previous research findings have shown that both patients and nurses typically rate the dimensions of “respecting patients” and “connecting with patients” relatively low (Moosavi et al., 2023; Okeny et al., 2024). Therefore, nursing administrators must prioritize enhancing nurses’ professional qualities and nurturing a caring culture within the nursing environment. Nurses should receive training in equitable patient treatment to foster respect and acceptance among patients. This strategy can effectively reduce hospital WPV incidents while improving nursing service quality and work efficiency.

The study also revealed that emotional stability negatively predicts WPV occurrence among nurses. Specifically, nurses exhibiting lower emotional stability are more susceptible to WPV. Previous studies have primarily examined the impact on care quality, professional life, and nurses’ emotional, psychological, and physical well-being (Liu et al., 2019b). This investigation illuminates how emotions influence individuals who have experienced WPV. Nurses scoring lower in emotional stability more frequently experience negative emotions such as anger, anxiety, and depression when addressing nurse–patient relationship challenges (Sattar et al., 2024). These individuals also demonstrate reduced emotional regulation and coping capabilities when responding to external stimuli. When nurses cannot effectively understand and address patient needs, communication breakdowns between nurses and patients or their families may trigger hostile reactions (Alnaeem et al., 2024). To develop more emotionally stable nursing professionals, personality trait training should be incorporated into nursing development programs. Such training should help emotionally unstable nurses understand their personality characteristics, recognize their impact on clinical practice, and develop optimism, positivity, and composure. In some instances, reassignment to departments with reduced direct patient contact may be considered for nurses with limited emotional regulation and coping abilities.

The relationship between WPV and openness reveals significant insights. Nurses scoring higher on the openness dimension demonstrate increased susceptibility to WPV experiences. This correlation can be attributed primarily to their enhanced capacity for establishing emotional connections with patients. While these nurses possess strong emotional engagement abilities, patients may misinterpret these connections, potentially leading to inappropriate responses and elevated risks of violent incidents (Gu et al., 2025). A descriptive investigation examining the correlation between nurses’ interpersonal approaches and assault incidents revealed that violent episodes predominantly occurred during patient needs assessment (Bilgin, 2009). Additionally, nurses exhibiting higher openness scores typically demonstrate greater willingness to explore novel medical approaches and maintain transparent communication with patients (Erdenk and Altuntaş, 2017). However, this openness may inadvertently increase the potential for patient conflicts and misunderstandings, thereby elevating WPV exposure risk.

The findings are similar to previous studies (Sui and Cheng, 2019) that indicated a negative correlation between years of experience openness scores and nurses’ WPV exposure, although logistic regression analysis did not yield statistically significant associations. However, the inclusion of years of experience openness in the regression model produced statistically significant results. This outcome may be attributed to the implementation of PSM, which minimized baseline differences between high and low WPV frequency groups, enhancing the regression model’s robustness and predictive accuracy (Schober and Vetter, 2020). These findings suggest that management should strengthen training programs focusing on emotional engagement and effective patient communication to reduce misunderstandings. Hospital administration should also collaborate with nursing staff to establish clear professional boundaries and roles, minimizing inappropriate expectations. Moreover, promoting teamwork can help mitigate individual uncertainty effects. Additionally, nurses should enhance their self-protection awareness and develop skills for effective violence response and safety preservation.

The study has several limitations. First, as this study was a cross-sectional study, it could not determine the causal relationship between WPV and personality traits as well as caring behaviors. Therefore, it will be necessary to longitudinal studies to test the current conclusions. Second, the study used convenience sampling without accounting for hospitals’ economic levels and geographical distribution, which may result in insufficient representativeness of the sample. Future research could expand the selection scope to enhance the generalizability of the findings. Third, although the scales used were valid and reliable, the data obtained were based on self-reports. Future studies can use behavioral records (such as the frequency of nursing procedures) or third-party assessments (such as patient evaluations) for cross-validation, thereby enhancing the objectivity and reliability of the data. Finally, a major limitation is that PSM can only control for observed confounders, which may restrict the generalizability of the findings.

## 5 Conclusion

In this study, all the nurses reported having been attacked by patients at some point last year with 1,714 (65.3%) experiencing a medium to high level of frequency of violence. Workplace violence has emerged as a significant occupational risk for nursing professionals. Consequently, investigating specific factors influencing nurses’ exposure to workplace violence is crucial for developing targeted preventive strategies. The study identified strong correlations between WPV exposure among nursing staff and their levels of respect, connectedness, emotional stability, and openness. These findings emphasize the importance for nurse managers to consider individual personality traits and caring behaviors when developing customized violence prevention training programs.

## Data Availability

The raw data supporting the conclusions of this article will be made available by the authors, without undue reservation.
