# Correction: The impact of nurses' caring behaviors and personality traits on workplace violence

**DOI:** 10.3389/fpsyg.2025.1666335

**Published:** 2025-09-15

**Authors:** Hongjuan Chang, Xin Liu, Mengmeng Hu, Rui Zeng, Chun Zhang, Huanhuan Luo

**Affiliations:** ^1^School of Medicine, Wuhan University of Science and Technology, Wuhan, China; ^2^School of Medicine, Tarim University, Alaer, Xinjiang, China

**Keywords:** nurses, caring, personality traits, tendency score matching, impact

Author Xin Liu was erroneously spelled as Xing Liu.

The original version of this article has been updated.

Author Chun Zhang was erroneously assigned to the affiliation “The Third People's Hospital of Hubei Province, Wuhan, China.” Author Chun Zhang should be affiliated with “School of Medicine, Wuhan University of Science and Technology, Wuhan, China.”

The affiliation “The Third People's Hospital of Hubei Province, Wuhan, China” has now been removed from the article.

The original version of this article has been updated.

